# Reinforcement Learning‐Guided Long‐Timescale Simulation of Hydrogen Transport in Metals

**DOI:** 10.1002/advs.202304122

**Published:** 2023-12-07

**Authors:** Hao Tang, Boning Li, Yixuan Song, Mengren Liu, Haowei Xu, Guoqing Wang, Heejung Chung, Ju Li

**Affiliations:** ^1^ Department of Materials Science and Engineering Massachusetts Institute of Technology Cambridge MA 02139 USA; ^2^ Research Laboratory of Electronics Massachusetts Institute of Technology Cambridge MA 02139 USA; ^3^ Department of Physics Massachusetts Institute of Technology Cambridge MA 02139 USA; ^4^ Department of Nuclear Science and Engineering Massachusetts Institute of Technology Cambridge MA 02139 USA

**Keywords:** hydrogen diffusion, long‐timescale simulations, reinforcement learning

## Abstract

Diffusion in alloys is an important class of atomic processes. However, atomistic simulations of diffusion in chemically complex solids are confronted with the timescale problem: the accessible simulation time is usually far shorter than that of experimental interest. In this work, long‐timescale simulation methods are developed using reinforcement learning (RL) that extends simulation capability to match the duration of experimental interest. Two special limits, RL transition kinetics simulator (TKS) and RL low‐energy states sampler (LSS), are implemented and explained in detail, while the meaning of general RL are also discussed. As a testbed, hydrogen diffusivity is computed using RL TKS in pure metals and a medium entropy alloy, CrCoNi, and compared with experiments. The algorithm can produce counter‐intuitive hydrogen‐vacancy cooperative motion. We also demonstrate that RL LSS can accelerate the sampling of low‐energy configurations compared to the Metropolis–Hastings algorithm, using hydrogen migration to copper (111) surface as an example.

## Graphical abstract

Computer simulation of atomic diffusion provides important insight into the kinetics of materials, but such long‐timescale simulation requires high computational costs. This work develops a reinforcement learning method that accelerates the simulation of atomic diffusion in alloys by two orders of magnitude. The method adaptively updates model parameters and selects diffusion pathways to simulate transition kinetics or sample low‐energy configurations.

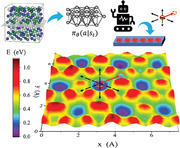

## 1 Introduction

Diffusive atomic motion is an essential microscopic process in the kinetic theory of materials.^[^ ^1^, ^2^ ^]^ Various interesting phenomena and applications are rooted in diffusion‐related processes, from the interdiffusion at metal interfaces, vacancy and void formation, to hydrogen embrittlement^[^ ^3^ ^]^ and resistance switching in oxide memristors.^[^ ^4^ ^]^ To investigate the diffusion process, atomic simulation^[^ ^5^, ^6^ ^]^ is often used to uncover atomic interactions behind a wide range of materials phenomena.^[^ ^2^, ^7^ ^]^ However, a critical challenge of atomistic simulation of diffusion‐related process is the timescale problem:^[^ ^8^ ^]^ the atomic vibration has a timescale of picoseconds, this limits the maximal time step that can be used in atomistic simulations; however, the diffusion‐related transitions between adjacent energy minima have orders of magnitude longer timescale. That is because the energy barriers on the diffusion pathway slow down the diffusion process.^[^ ^2^ ^]^ The timescale problem limits most of the straightforward molecular dynamics simulations to nanoseconds, which fall short of the timescales relevant to many diffusion‐related phenomena.^[^ ^8^, ^9^ ^]^ Therefore, different methods are needed to deal with the long‐timescale problem.^[^ ^8^ ^]^

Our work will be compared with one of the widely studied algorithms, the kinetic Monte Carlo (KMC) method,^[^ ^10^ ^]^ where one directly works with diffusion timescale without explicitly showing the vibration timescale motion. Traditional KMC (in contrast with off‐lattice KMC) requires energy minima and transition pathways (the so‐called event table) as input, and the method chooses transition events according to the transition rates in the event table. However, as the diffusion pathway is sometimes counter‐intuitive, correctly determining the necessary input information of KMC is not a trivial task.^[^ ^10^ ^]^ To conduct a simulation without a known event table, the off‐lattice KMC is developed.^[^ ^11^ ^]^ The algorithm conducts saddle‐point searches to obtain the diffusion pathways along with the KMC simulation. Another method reported to have advantageous efficiency is temperature accelerated dynamics (TAD), where the transition pathways are explored by high‐temperature molecular dynamics.^[^ ^12^ ^]^ In both methods, the transition pathway is explored by random sampling (random initial guess in the saddle‐point search for off‐lattice KMC, and random thermal motion for TAD). However, as the configuration space is high dimensional, it requires a large amount of random sampling to ensure that the correct transition pathway is obtained, which limits the simulation system size and accessible timescale.^[^ ^11^ ^]^

In this work we developed a reinforcement learning (RL) based method that guides the transition pathway sampling on chemically complex potential energy surface (PES). Instead of searching for all nearby saddle points along randomly sampled initial directions,^[^ ^11^ ^]^ we use parameterized neural network model to predict the direction of atomic motion that yields the high‐probability transition pathway, based on learning from the outcomes of rigorous PES minimum energy path (MEP) searches, resulting in a data superstructure of reduced‐dimension “transition energy landscape” (TEL). The neural network based TEL avoids the repeated saddle‐point searches, which is the most significant contributor to the computational cost of the off‐lattice KMC. We demonstrate that our RL model can either simulate physical time diffusional trajectories (RL TKS) or sample low‐energy configurations (RL LSS) efficiently, in various hydrogen diffusion phenomena in alloy bulk or near surfaces.

## 2 Results

### 2.1 General Framework

#### 2.1.1 Computational Workflow

**Figure 1:**
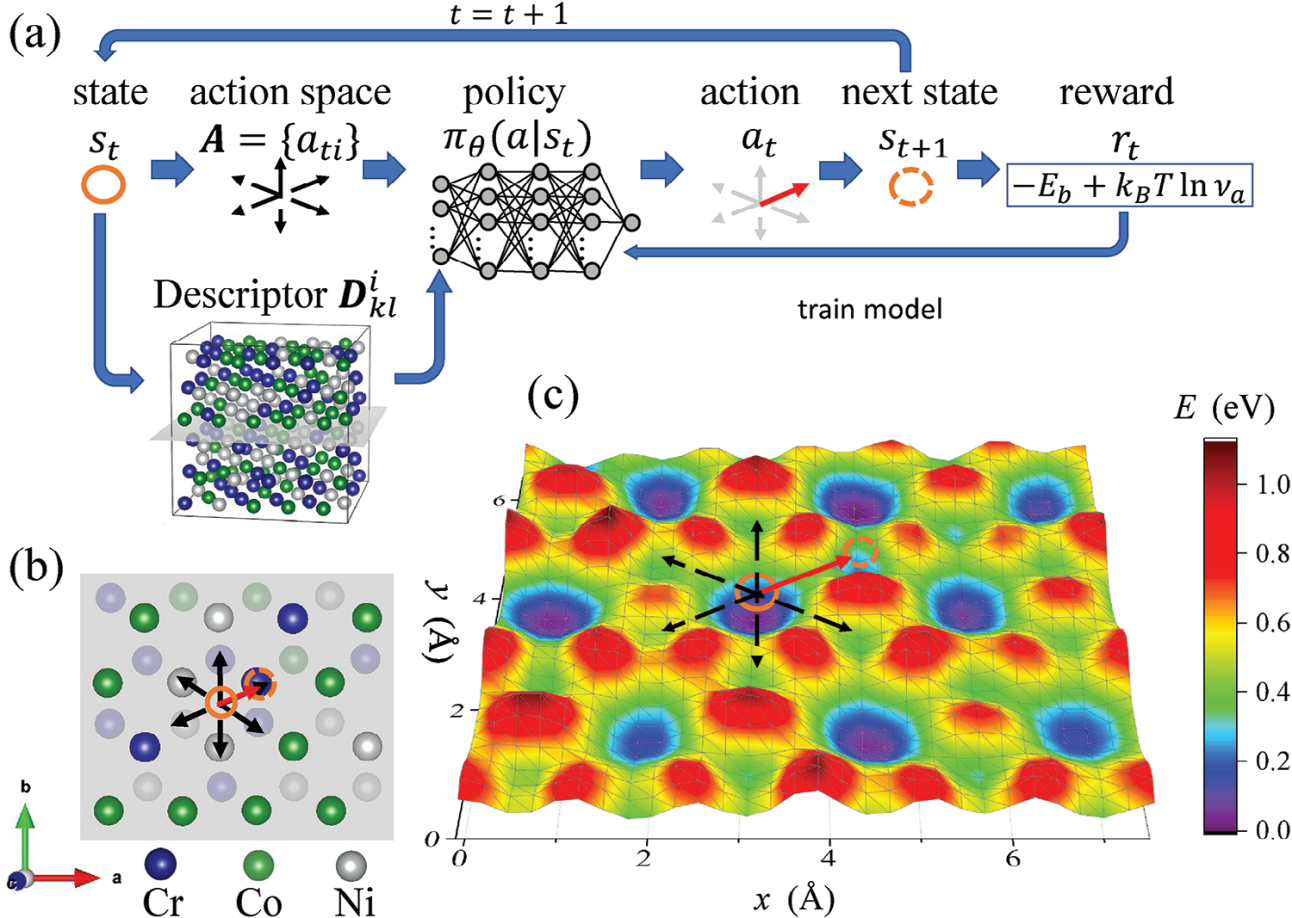
a) Computational workflow of the RL long‐timescale method illustrated on b) hydrogen diffusion in CrCoNi medium entropy alloy. The blue, green, and grey spheres represent Cr, Co, and Ni atoms, respectively. The orange circle, black dashed arrow, and red arrow represent state, action space, and selected action, respectively. c) The potential energy landscape of a hydrogen atom on the grey planes in (a, b). When calculating the energy, surrounding atoms and the *z*‐coordinate of the hydrogen atoms are relaxed.

Our RL method is illustrated in **Figure** **1**a. The PES has a large number of local minima separated by transition energy barriers. In this paper, we use hydrogen diffusion in face‐centered cubic (FCC) alloys as an example, as shown in Figure 1b. In the local energy minimum configurations of FCC bulk structures, hydrogen atoms can reside in octahedral and tetrahedral interstitial sites shown as the deep blue and shallow green potential wells in Figure 1c, and the octahedral site corresponds to a lower‐energy configuration. The energy landscape is provided by a universal neural network interatomic potential (which evaluates the total energy and forces on atoms for a given atomic configuration), the PreFerred Potential (PFP),^[^ ^13^, ^14^ ^]^ throughout this paper. Beginning from a given local energy minimum configuration or “state” $s_{t}\equiv \left({\vec{r}}_{1},{\vec{r}}_{2},\text{…},{\vec{r}}_{N}\right)$ (the orange circles in Figure 1, where ${\vec{r}}_{i}$ are the coordinates of the *i*th atom), a set of possible transition displacements {*a*_*ti*_} (also called “actions”) are first identified. In our problem, we first identify the polyhedron formed by the nearest‐neighbor metal atoms of each interstitial hydrogen. Possible actions are then defined by moving the hydrogen atom through the face centers of the polyhedron (See Section 4.1 for details).

In the next step, an action *a*_*t*_ is selected from the action space $A_{s_{t}}\equiv \left\{a_{ti}\right\}$. The probability of selecting each action *a* given current state *s*_*t*_ is defined as π_θ_(*a*|*s*_*t*_), which is given by the Boltzmann policy based on a neural network value function *Q*_θ_(*s*_*t*_, *a*).^[^ ^15^ ^]^

(1) $$\pi_{\theta }\left(a|s_{t}\right)=\frac{e^{Q_{\theta }\left(s_{t},a\right)/k_{B}T}}{\sum_{a^{\prime }\in A_{s_{t}}}e^{Q_{\theta }\left(s_{t},a^{\prime }\right)/k_{B}T}}$$

where *Q*_θ_(*s*, *a*) represents the expected reward that can be obtained by taking a specific action *a* in a particular state *s*, and the actions with higher expected rewards are more likely to be selected (Equation (1)). The Boltzmann form reflects the randomness caused by temperature effects. θ represents the neural‐network model parameters, *k*_B_ and *T* are the Boltzmann constant and physical temperature. We note that *Q*_θ_(*s*, *a*) itself can also depend on *T* if the vibrational entropy contribution is considered, which will be discussed later. While Equation (1) looks very similar to the well‐known KMC dynamics, the meaning of *Q*_θ_(*s*_*t*_, *a*) is quite different, in that *Q*(*s*_*t*_, *a*) reflects not only information about the present (i.e., the present‐step forward energy barrier), but also “future returns” that involve *future* energy barriers and/or *future* free‐energy reductions, in a combination that is to be detailed later. *Q*_θ_ is neural network fitting of *Q*, where θ stands for the set of neural network parameters, which is the convention in this paper. Because of this conceptual distinction with KMC, the “dynamics” generated by Equation (1) is not guaranteed yet to be the real physical timescale according to transition‐state theory (TST), as KMC aims to. And so the time label *t* in *s*_*t*_, *a*_*t*_ above is an integer, and not the real time yet.

After selecting an action $a_{t}=\left(i,\vec{v}\right)$ (both *i* and $\vec{v}$ are determined by *a*_*t*_), the *i*th atom is displaced by vector $\vec{v}$ across the energy barrier. The system is then relaxed to the next state, *s*_*t* + 1_, using the MDMin algorithm implemented in the Atomistic Simulation Environment.^[^ ^16^ ^]^ Parameters of the transition, including the transition energy barrier $E_{b}^{\text{NEB}}$, the attempt frequency $\nu_{a}^{\text{label}}$, and the energy change after the transition Δ*E*, can then be computed using PFP. The transition saddle point is obtained by the nudged elastic band (NEB) method^[^ ^17^ ^]^ by setting *s*_*t*_ and *s*_*t* + 1_ as the initial and final points, to provide the ground truths for neural network training. The reward function of this transition step, *r*_*t*_, the key concept in RL, can take different forms depending on the goal of the RL dynamics (specified in Equation (1)) which will be detailed later. The whole simulation trajectory is produced by repeating the above scheme that generates the next state according to the current state.

#### 2.1.2 Model Architecture of *Q*_θ_(*s*, *a*)

The *Q*_θ_(*s*, *a*) model is constructed based on the DeepPot‐SE sub‐networks.^[^ ^18^ ^]^ As the atomic interaction in alloys is short ranged, we assume $Q_{\theta }\left(s,a=\left(i,\vec{v}\right)\right)$ is a function of the atomic environment of the moved atom *i* and its displacement vector $\vec{v}$. The function *Q*_θ_(*s*, *a*) should be invariant under translation, rotation, and permutation operations on the atomic system. Therefore, we define an atomic descriptor $D^{i}$, which is a re‐formalization of *s* and *a*, that is invariant under all symmetry operations. $D^{i}$ can be realized by the following construction.

(2) $${\tilde{R}}^{i}=\left[\begin{array}{cccc} {\hat{\vec{r}}}_{i1}\cdot {\hat{\vec{r}}}_{i1} & \cdots & {\hat{\vec{r}}}_{i1}\cdot {\hat{\vec{r}}}_{iM} & {\hat{\vec{r}}}_{i1}\cdot \vec{v}\\ {\hat{\vec{r}}}_{i2}\cdot {\hat{\vec{r}}}_{i1} & \cdots & {\hat{\vec{r}}}_{i2}\cdot {\hat{\vec{r}}}_{iM} & {\hat{\vec{r}}}_{i2}\cdot \vec{v}\\ \vdots & \vdots & & \vdots \\ {\hat{\vec{r}}}_{iM}\cdot {\hat{\vec{r}}}_{i1} & \cdots & {\hat{\vec{r}}}_{iM}\cdot {\hat{\vec{r}}}_{iM} & {\hat{\vec{r}}}_{iM}\cdot \vec{v}\\ \vec{v}\cdot {\hat{\vec{r}}}_{i1} & \cdots & \vec{v}\cdot {\hat{\vec{r}}}_{iM} & |\vec{v}{|}^{2} \end{array}\right]$$

(3) $$D_{kl}^{i}=\sum_{m,n=1}^{M+1}G_{k}^{1}\left(f_{c}\left(r_{im}\right),c_{m}\right){\tilde{R}}_{mn}^{i}G_{l}^{2}\left(f_{c}\left(r_{in}\right),c_{n}\right)$$

where ${\hat{\vec{r}}}_{ij}\equiv \frac{f_{c}\left(r_{ij}\right){\vec{r}}_{ij}}{r_{ij}},{\vec{r}}_{ij}\equiv {\vec{r}}_{j}-{\vec{r}}_{i}$, $r_{ij}\equiv \left|{\vec{r}}_{ij}\right|$, *j* = 1, 2, ⋅⋅⋅, *M* goes through all atoms around the *i*th atom within a cut‐off radius *r*_c_. The summation in Equation (3) goes from 1 to *M* + 1 to go through all rows/columns in the matrix in Equation (2). *f*_c_(*r*) is a cut‐off function as defined in ref. [18], which goes smoothly to zero at a cut‐off radius *r*_c_, and $G_{k}^{1}$ and $G_{l}^{2}$ are embedding neural networks parametrized by θ_emb_. *c*_*m*_(*m* = 1, 2, ⋅⋅⋅, *M*) are the atomic species of the *m*th atom. To embed the action (the last row and column in Equation (2)) into the descriptor, we set *c*_*M* + 1_ as a unique “action species.” The descriptor $D^{i}$ is invariant under all symmetry operations. The descriptor is then flattened to a vector and passed to a multilayer perceptron (MLP) that outputs the *Q* function: $Q_{\theta }\left(s,a=\left(i,\vec{v}\right)\right)={\mathrm{MLP}}_{\theta_{\mathrm{fit}}}\left(D^{i}\left(\theta_{\mathrm{emb}}\right)\right)$, where the model parameters θ = (θ_fit_, θ_emb_) include both parameters of the MLP θ_fit_ and that of the embedding network θ_emb_ (see Section S1, Supporting Information for detailed parameter settings).

By choosing different reward functions *r*_*t*_ (whose accumulated form becomes *Q* in standard RL formalism, for Equation (1)), our method has at least two working modes: transition kinetics simulator (TKS) and low‐energy states sampler (LSS). RL TKS aims to simulate physical transition rates according to the HTST (thus is in principle identical to KMC, just with neural network estimators, and so the *t* label aims to lead to real physical time also), while the LSS aims to converge to global energy minimum configurations and is thus similar to an energy annealer, where the *t* is fictitious. In the next two sections we will introduce the two special options, RL TKS and RL LSS, separately. In Section 4.3, we will also discuss the mathematical and physical meaning of general RL (finite α, β, γ), away from the TKS (α = 1, β = 0, γ = 0) and LSS (α = 0, β = 1, γ ≈ 1) corners in parameter space.

### 2.2 RL Transition Kinetics Simulator Option

TKS adopts the reward function of

(4) $$r_{t}^{\mathrm{TKS}}\equiv -E_{b}^{\mathrm{NEB}}+k_{B}T\ln\nu_{a}^{\mathrm{label}},\;\;\;\nu_{a}^{\mathrm{label}}=\frac{\prod_{i=1}^{3M}\nu_{i}}{\prod_{j=1}^{3M-1}\nu_{j}^{*}}$$

where the $E_{b}^{\mathrm{NEB}}$ and $\nu_{a}^{\mathrm{label}}$ are obtained using the PFP on the fly during the neural network training. The attempt frequency $\nu_{a}^{\mathrm{label}}$ in our training data is evaluated using ν_*i*_ and $\nu_{j}^{*}$, the *i*th normal mode vibrational frequency at state *s*_*t*_ and the *j*th positive vibrational frequency at the PES saddle point between *s*_*t*_ and *s*_*t* + 1_. (The first‐order saddle point has one non‐positive mode, so *j* only runs from 1 to 3*M* − 1 excluding the non‐positive mode, while *i* runs from 1 to 3*M*.) We calculate ν_*i*_ and $\nu_{j}^{*}$ by diagonalizing the force constant matrix evaluated by the PFP for the *M* atoms within a cut‐off radius *r*_c_ = 4 Å from the atom displaced by the action.

The model is trained as a contextual bandit problem,^[^ ^19^ ^]^ where the value function $Q_{\theta }^{\mathrm{TKS}}\left(s_{t},a_{t}\right)$ is trained to fit the instantaneous reward $r_{t}^{\mathrm{TKS}}$ (minimizing $\left\langle {\left(Q_{\theta }^{\mathrm{TKS}}\left(s_{t},a_{t}\right)-r_{t}^{\mathrm{TKS}}\right)}^{2}\right\rangle$). This means that we have set the RL discount‐rate γ = 0, so the RL value function *Q* is no longer cumulative or inclusive of the future rewards and cares only about the present‐step immediate reward. Furthermore, this immediate reward is taken to be just the vibrational free‐energy barrier of the forward transition in harmonic transition state theory (HTST), which means TKS is just a version of KMC, but with neural network learning for acceleration.

To associate an action with a physical time scale, the transition rate can be evaluated by $Q_{\theta }^{\mathrm{TKS}}$ according to HTST:

(5) $$\Gamma_{s_{t}a}=\nu_{a}^{\mathrm{label}}e^{-E_{b}^{\mathrm{NEB}}/k_{B}T}=e^{r_{t}^{\mathrm{TKS}}/k_{B}T}≃e^{Q_{\theta }^{\mathrm{TKS}}\left(s_{t},a\right)/k_{B}T}$$

Equation (1) then gives the physical branching ratio (probability) $P\left(a|s_{t}\right)=\Gamma_{s_{t}a}/\sum_{a^{\prime }\in A_{s_{t}}}\Gamma_{s_{t}a^{\prime }}$. The average residence time of the system on the state *s*_*t*_, $\left\langle \Delta \tau \right\rangle ={\left(\sum_{a\in A_{s_{t}}}\Gamma_{s_{t}a}\right)}^{-1}$, can be estimated using the $Q_{\theta }^{\mathrm{TKS}}$ functions as

(6) $$\tau =1/\sum_{a\in A_{s_{t}}}e^{Q_{\theta }^{\mathrm{TKS}}\left(s_{t},a\right)}$$

Note that the *t* symbol is an integer in RL, counting the number of state transitions, so *t* is not the physical time, which are denoted by τ in this paper instead.

Then, we make the model applicable to different temperatures. Expressing the reward $r_{t}^{\mathrm{TKS}}=r_{t}^{0}+r_{t}^{1}T$ as a linear function of *T*, the zeroth‐order term $r_{t}^{0}$ and the linear term $r_{t}^{1}$ can be fitted simultaneously by a two‐component value function $\left(Q_{\theta }^{0},Q_{\theta }^{1}\right)$ in $Q_{\theta }^{\mathrm{TKS}}=Q_{\theta }^{0}+Q_{\theta }^{1}T$.

(7) $$\theta \leftarrow \theta -\lambda \nabla_{\theta }\sum_{t}\left[{\left(Q_{\theta }^{0}\left(s_{t},a_{t}\right)-r_{t}^{0}\right)}^{2}+T_{\text{tr}}^{2}{\left(Q_{\theta }^{1}\left(s_{t},a_{t}\right)-r_{t}^{1}\right)}^{2}\right]$$

where λ is the learning rate, and *T*_tr_, the training temperature, is a hyperparameter that controls the relative importance of the two terms in the loss function (which does not need to be the physical temperature in simulations). By introducing temperature into $Q_{\theta }^{\mathrm{TKS}}$, $Q_{\theta }^{0}$, and $Q_{\theta }^{1}$ give neural network predictions for the energy barrier $E_{b}^{\text{NN}}\equiv -Q_{\theta }^{0}$ and attempt frequency $\log\nu_{a}^{\text{NN}}\equiv \frac{Q_{\theta }^{1}}{k_{B}}$.

As a testbed, we first apply RL TKS to hydrogen diffusion in pure FCC Cu and Ni. The model is trained on a 4 × 4 × 4 cubic supercell with four randomly sampled hydrogen sites. The model is then deployed to simulate a single hydrogen diffusion in a 3 × 3 × 3 cubic supercell for 500 timesteps. This system is simulated by RL TKS at temperatures spanning 250 to 500 K with an interval of 50 K and repeated 50 times for each temperature. The time‐dependent squared displacements $\Delta x_{j}^{2}\left(\tau \right)$ and temperature *T*_*j*_ of each (*j*th) simulation trajectory are recorded. The diffusivity *D*(*T*) for each given temperature *T* is extracted from the linear fitting ${\left\langle \Delta x_{j}^{2}\left(\tau \right)\right\rangle }_{T_{j}=T}=6D\left(T\right)\tau$. The two parameters *D*_0_ and *E*_a_ in the Arrhenius form of diffusivity $D=D_{0}e^{-E_{a}/k_{B}T}$ are derived from the $\ln D(T)=\ln D_{0}-\frac{E_{a}}{k_{B}}\frac{1}{T}$ fit.

First, we checked that our RL TKS are consistent with the simulation results of traditional KMC using the same PFP interatomic potential, as shown in **Table** **1**. This validates our RL methods in estimating hydrogen diffusivity in metals. The derived *D*_0_ and *E*_a_ are also reasonably consistent with the related experimental measurements. The effective activation energy *E*_a_ in simulation tends to be slightly smaller than the experimental results for multiple reasons. First, the PFP machine learning potential we used tends to slightly underestimate the energy barrier. For example, in FCC copper, the diffusion energy barriers in O →T/T → O (O → T means from octahedral to tetrahedral, and T → O means the reverse process) transition are 0.32/0.12 eV using the PFP compared with 0.36/0.14 eV in the DFT calculations.^[^ ^20^ ^]^ Second, the quantum tunneling effects in H diffusion can further influence the activation barrier, which is not considered in our classical dynamics calculations. The O → T activation barrier in FCC copper considering quantum tunneling is estimated as 0.40 eV at 300 K using the path‐integral Monte Carlo method, 0.04 eV higher than that without considering the quantum tunneling effect.^[^ ^20^ ^]^ Therefore, our calculation here slightly underestimate activation energies. As a PES transition dynamics sampling algorithm, our RL method is compatible with different methods to estimate activation barriers. Using the DFT or PIMD method to calculate activation barriers in our training dataset will improve the prediction accuracy.

**Table 1:** RL TKS hydrogen self‐diffusion simulation results in pure copper, pure nickel, and CrCoNi medium entropy alloy. $\Delta E_{b}\equiv \sqrt{\left\langle {\left(E_{b}^{\mathrm{NN}}-E_{b}^{\mathrm{NEB}}\right)}^{2}\right\rangle }$ and $\Delta \nu_{a}\equiv \sqrt{\left\langle {\left(\ln\nu_{a}^{\mathrm{NN}}-\ln\nu_{a}^{\mathrm{label}}\right)}^{2}\right\rangle }$ are the validation error of model prediction on transition energy barrier and attempt frequency. The activation energy *Q* and coefficient *D*_0_ are fitted by reinforcement‐learning‐simulated diffusivity $D=D_{0}e^{-E_{a}/k_{B}T}$ using maximal‐likelihood estimation, and $D_{0}^{\text{exp}}$ and $E_{a}^{\text{exp}}$ are the values from previous experiments.

	Cu	Ni	CrCoNi
Δ*E*_b_ (eV)	0.020	0.022	0.037
Δln ν_a_	0.09	0.12	0.12
*D*_0_(10^−7^m^2^/s)	3.6	3.1	5
*E*_a_(eV)	0.30	0.33	0.43
$D_{0}^{\mathrm{KMC}}\left(10^{-7}m^{2}/s\right)$	3.3	2.8	–
$E_{a}^{\mathrm{KMC}}\left(\mathrm{eV}\right)$	0.31	0.32	–
$D_{0}^{\text{exp}}\left(10^{-7}m^{2}/s\right)$	3.69^[^ ^21^ ^]^	0.15–6.98^[^ ^22^ ^]^	–
	21.1^[^ ^23^ ^]^	1.1–6.87^[^ ^24^ ^]^	–
	17.4^[^ ^25^ ^]^		
$E_{a}^{\text{exp}}\left(\mathrm{eV}\right)$	0.38^[^ ^21^ ^]^	0.31–0.44^[^ ^22^ ^]^	–
	0.46^[^ ^23^ ^]^	0.37–0.44^[^ ^24^ ^]^	–
	0.435^[^ ^25^ ^]^		

**Figure 2:**
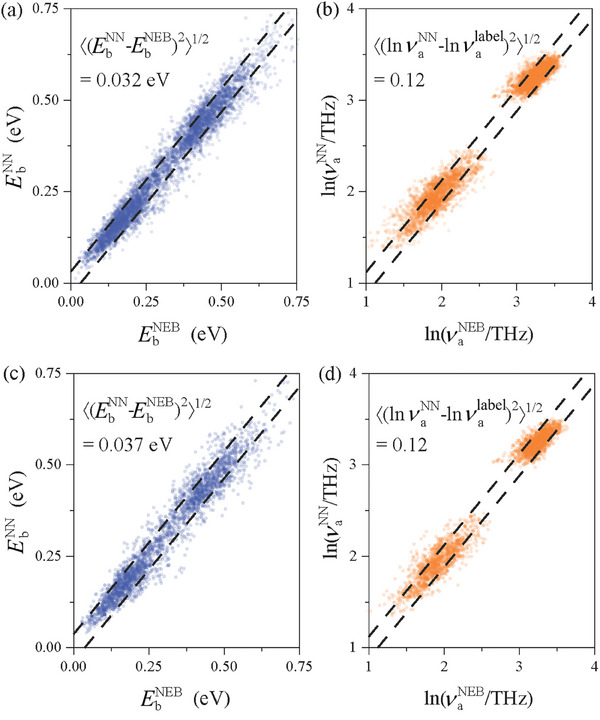
Comparison of the TEL neural network prediction of a) transition energy barriers $E_{b}^{\mathrm{NN}}$ and b) attempt frequency $\nu_{a}^{\mathrm{NN}}$ with those calculated by the NEB method in the training dataset, $E_{b}^{\mathrm{NEB}}$ and $\nu_{a}^{\mathrm{label}}$. Here we show the data for hydrogen diffusion in equiatomic CrCoNi alloy. Validation on the testing dataset is shown in c) and d).

To test the method's capability to capture chemical complexity, we train the RL model on equiatomic CrCoNi medium entropy alloy. The CrCoNi alloy has recently attracted interest because of its outstanding fracture toughness and ductility.^[^ ^26^ ^]^ In the CrCoNi solid solution, each metal atom near the hydrogen can be of different atomic species, giving a complex atomistic state space. The predicted $E_{b}^{\mathrm{NN}}$ and $\nu_{a}^{\mathrm{NN}}$ are approximately consistent with the values in the training and testing datasets, as shown in **Figure** **2**, where the data points are distributed close to the diagonal line in the wide range of observed quantities. The standard deviation errors of the model predictions are close in training and testing datasets, confirming that the training data is not overfitted despite the large number of neural network parameters.

**Figure 3:**
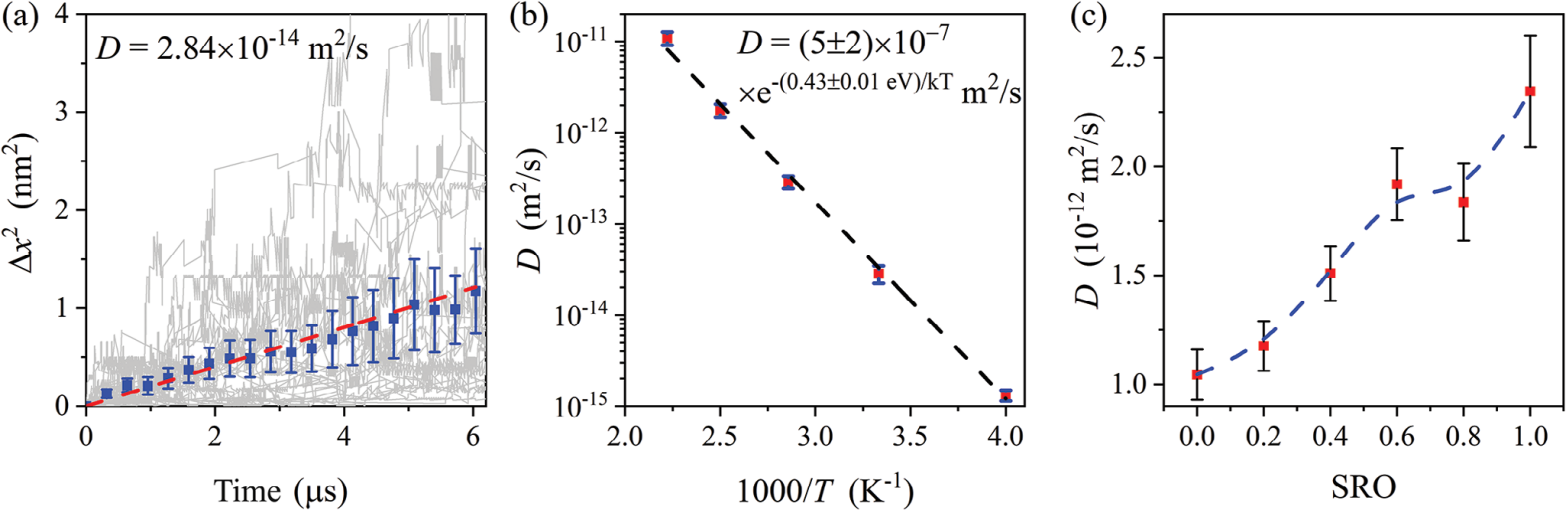
RL TKS hydrogen diffusion simulation in CrCoNi medium entropy alloy. a) Square hydrogen diffusion displacement Δ*x* ^2^ (absolute value) as a function of time under 300 K. The grey lines show 30 trajectories; the blue squares and error bars are the mean square displacements and their error range (± one standard error); the red dashed line is the linear fitting of the blue dots. b) Arrhenius plot of hydrogen self‐diffusivity under different temperatures. The blue caps show the error bar of calculated diffusivities, and the black dashed line is a linear fitting of log *D* versus $\frac{1000}{T}$. c) Hydrogen self‐diffusivity at 400 K as a function of the short‐range ordering parameter (the dashed line is a B‐spline^[^ ^28^ ^]^ connecting the data points). SRO = 0 corresponds to a fully random solid solution, SRO = 1 corresponds to WC parameters obtained from ref. [27], and intermediate values of SRO are linearly interpolated. The SRO is sampled using the OTIS code in ref. [29].

The hydrogen self‐diffusion in CrCoNi is simulated using RL TKS running on one hydrogen in a 4 × 4 × 4 rhombohedral supercell with short‐range ordering (SRO) obtained from ref. [27]. The hydrogen squared displacement as a function of the RL TKS simulation time is shown in **Figure** **3**a under 300 K using 30 repetitions of µs long‐timescale simulations. An approximate function form of 〈Δ*x* ^2^〉∝τ is shown by the blue line, and the diffusivity is estimated to be 2.84 × 10^−14^ m^2^ s^−1^. Similar simulations are implemented for different temperatures, as shown in Figure 3b. The Arrhenius plot shows a good linear relation. The estimated effective activation energy *E*_a_ equals 0.43 ± 0.01 eV, and the pre‐exponential factor *D*_0_ equals 5 ± 2 × 10^−7^ m^2^ s^−1^. To our knowledge, these parameters have not been provided in the literature, so we show these results as predictions of our method.

In CrCoNi, SRO has significant influences on various properties of the material ranging from hardness^[^ ^30^ ^]^ and stacking fault energy^[^ ^27^ ^]^ to magnetism.^[^ ^31^ ^]^ We show that the SRO also has an evident influence on the hydrogen diffusivity in CrCoNi, as shown in Figure 3c. The system with SRO under thermal equilibrium (SRO = 1) gives approximately double the hydrogen diffusivity of the fully random configuration (SRO = 0), showing that the SRO enhances hydrogen diffusion. This can be explained by the reduction of Cr–Cr bond concentration by the SRO,^[^ ^27^ ^]^ as the hydrogen transition energy barriers proximate to the Cr–Cr bond are found to be higher than the average hydrogen transition energy barriers. Our results predict that the hydrogen diffusion behavior can also be tuned by the SRO in multi‐principal element alloys.

**Figure 4:**
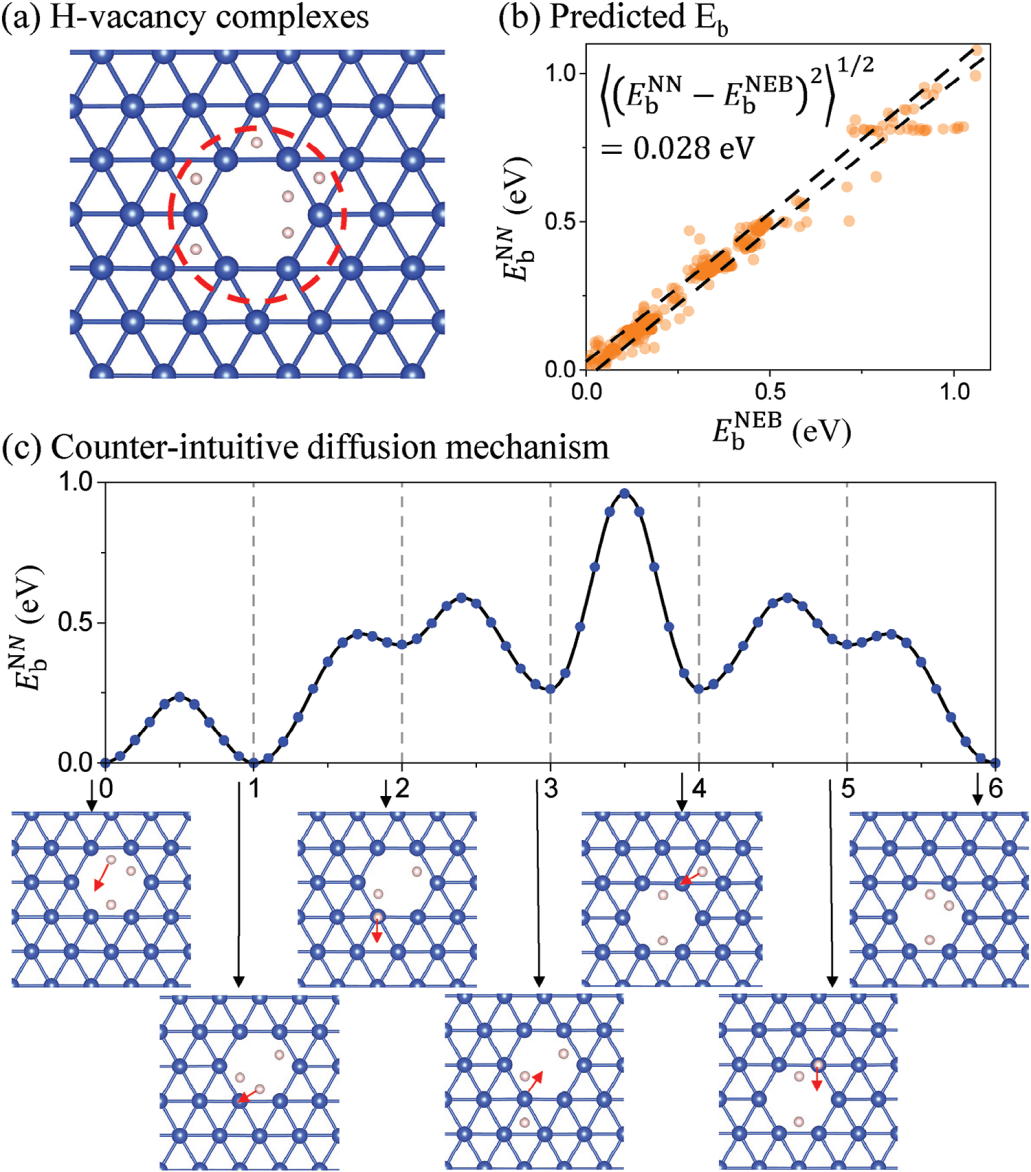
Hydrogen‐vacancy complexes diffusion in FCC copper with RL TKS. a) Atomic configuration of a hydrogen‐vacancy complex, where multiple hydrogen atoms are adsorbed around a vacancy site. The blue and pink spheres represent copper and hydrogen atoms, respectively. b) NN predicted energy barriers compared with the energy barriers calculated by the NEB method. c) A counter‐intuitive diffusion mechanism identified from the RL TKS simulation.

RL TKS can also be used to discover geometrically surprising diffusion mechanisms, where the diffusion pathway can be counter‐intuitive and involve cooperative motion of multiple atoms. We apply our method to the hydrogen‐vacancy (HV) complexes diffusion in FCC copper.^[^ ^32^ ^]^ The HV complex consists of a copper vacancy and a few hydrogen atoms adsorbed around the vacancy, as shown in **Figure** **4**a. RL TKS is trained on a series of HV complexes containing one to eight adsorbed hydrogen atoms, providing a prediction accuracy of 28 meV for *E*_b_ (Figure 4b) and 0.10 for ln ν_a_. We use RL TKS to simulate the HV complex diffusion, which can happen through multiple different transition pathways. Here, we present the transition pathway of one frequently appearing diffusion event, shown in Figure 4c. As the hydrogen distribution influences the vacancy transition rate, one hydrogen atom moves ahead to form a hydrogen arrangement that enhances the transition rate of the vacancy (step 0–3). The vacancy then follows the hydrogen (step 3–4). Finally, the hydrogen left behind follows the vacancy, completing the overall translation of the complex. Such a complex diffusion mechanism can hardly be conjectured by human, showing that our method can be applied to cases when the KMC event table is hard to construct without deep learning (in our case, the reduced‐dimension “transition energy landscape” TEL is constructed based on Equations (2) and (3), and see also Section 4.1).

**Figure 5:**
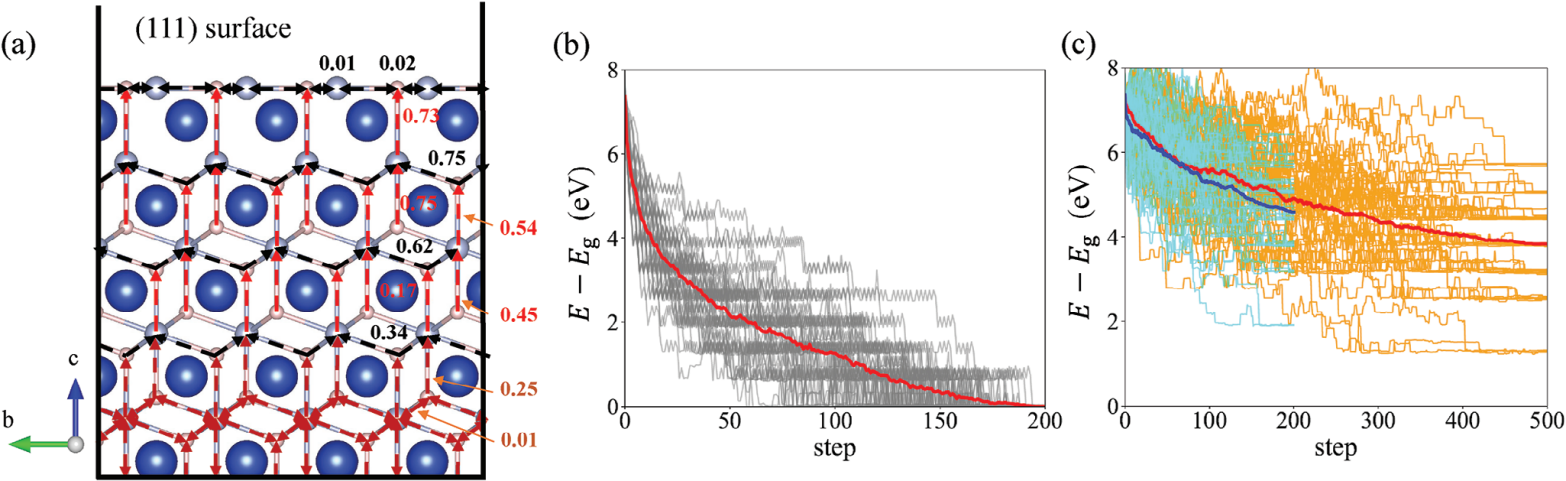
RL LSS sampling low‐energy configurations of hydrogen migration to copper (111) surface. a) Highest probability actions (HPAs) and *Q* values of hydrogen atoms. The blue, silver, and pink spheres are copper atoms, octahedral interstitial sites, and tetrahedral interstitial sites. The HPAs (the actions with the highest probability according to the policy) are shown by arrows (red arrow: a unique HPA, black arrow: multiple [but not all] actions with equal probabilities, brown arrow: all actions have equal probabilities). The *Q* values of HPAs are denoted. Energy (using ground state energy as reference) versus simulation step under simulated annealing with b) $T=1000-950\frac{t}{200}K$ using the trained policy and c) $T=3000-2700\frac{t}{\tau_{\mathrm{anneal}}}K$ (τ_anneal_=200 for blue lines and τ_anneal_=500 for red lines) using Metropolis–Hastings algorithm. The grey/cyan/orange thin lines are 50 simulation trajectories, and the thick red/blue lines are their average.

RL TKS provides evident speed‐up compared to off‐lattice KMC without RL acceleration, as shown in **Figure** **5** in Section 4.2. Although implementing the TKS requires RL training in advance, their simulation runtime per transition step is about two orders of magnitudes smaller than off‐lattice KMC. Therefore, the overall computational costs of the RL methods are smaller than the off‐lattice KMC as long as the total number of simulation steps is larger than the threshold. We can see that our simulations in Figure 3 involve far more simulation steps than the threshold, demonstrating that RL methods provide significant acceleration. The acceleration is because to determine transition probabilities, one needs to do expensive saddle‐point searches for all possible actions in the off‐lattice KMC, while we just need to evaluate the *Q*_θ_ function in RL TKS, which is much faster.

### 2.3 RL Low‐Energy States Sampler Option

The second option of our method, LSS, is a “true” RL method in that the discount‐rate γ is generally taken to be finite and positive, so the RL value function *Q* is cumulative and inclusive of future rewards. That is to say, LSS looks long into the future event horizon. LSS also typically sets the reward function as the energy reduction after the transition:

(8) $$r_{t}^{\mathrm{LSS}}\equiv E\left(s_{t}\right)-E\left(s_{t+1}\right)$$

(*E*(*s*) is the potential energy of state *s*). We may also include the vibrational free‐energy contribution

(9) $$r_{t}^{\mathrm{LSS}}\equiv F\left(s_{t}\right)-F\left(s_{t+1}\right)$$

if we choose to, which would involve the extra computational cost of computing and diagonalizing the Hessian matrices on the fly, or its learned version

(10) $$r_{t}^{\mathrm{LSS}}\equiv F_{\theta }\left(s_{t}\right)-F_{\theta }\left(s_{t+1}\right).$$

The model is trained by the Deep Q‐Network (DQN) algorithm,^[^ ^33^ ^]^ which aims to maximize the total reward

(11) $$R^{\mathrm{LSS}}\equiv \sum_{t=0}^{t_{\mathrm{horizon}}-1}\gamma^{t}r_{t}^{\mathrm{LSS}}$$

on a trajectory with a discount factor γ close to one (set as 0.8 in our calculation). The model parameters are updated according to the Bellman equation.^[^ ^33^ ^]^

(12) $$\theta \leftarrow \theta -\lambda \nabla_{\theta }\sum_{t}{\left(r_{t}^{\mathrm{LSS}}+\gamma \max_{a^{\prime }}Q_{\theta^{t}}^{\mathrm{LSS}}\left(s_{t+1},a^{\prime }\right)-Q_{\theta }^{\mathrm{LSS}}\left(s_{t},a_{t}\right)\right)}^{2}$$

where θ^*t*^ is the target network that updates less frequently than θ, and then one intermittently assigns θ to θ^*t*^ to iterate. Such training gradually builds up the TEL neural network, in the reduced‐dimension action space $A\left(s\right)=\{a=\left(i,\vec{v}\right)\}$ based on the atomic configuration *s*, which is much smaller than the 3*N*‐dimensional PEL and is highly adaptive, that is, it is trained to pay “attention” to only the small subspace of *s* that is likely to lead to large *Q* within time horizon *t*_horizon_.

The converged $Q_{\theta }^{\mathrm{LSS}}\left(s_{t},a_{t}\right)$ represents reduced‐dimension TEL and fits the expected value of the maximal total rewards after timestep *t*, $\max_{\left(a_{t+1},a_{t+2},\text{…}\right)}\sum_{t^{\prime }=t}^{t_{\mathrm{horizon}}-1}\gamma^{t^{\prime }-t}r_{t^{\prime }}^{\mathrm{LSS}}$ (interested readers can find details about the DQN training algorithm and implementation in RL textbooks). As the *Q* ^LSS^ function “foresees” the energy reduction of future steps and chooses actions that maximize long‐term cumulative return, it is expected to converge to low‐energy configurations faster than local strategies that only consider single‐step energy terms. RL LSS is thus an efficient annealer (“the end justifies the means”), which converges to a near‐ground state with fewer timesteps than RL TKS (“procedural justice”).

We demonstrate RL LSS's performance in simulating energy annealing by the process of hydrogen migration to copper (111) surface, as shown in Figure 5a. 4 × 4 × 3 hexagonal supercells are constructed with 10 randomly sampled hydrogen sites, and the (111) surface is created with a 15 Å vacuum layer. Hydrogen in the surface adsorption sites has lower energy than that in the bulk interstitial site, so the energy ground state is that all hydrogen atoms are on the surface adsorption sites. However, because of the energy difference between the octahedral sites and tetrahedral sites, the migration pathway involves multiple local energy minimums and low‐energy barriers, making it challenging to sample the lowest‐energy states.^[^ ^34^ ^]^ After training, our RL policy gives the most likely action from each state, as shown in Figure 5a. Within the cut‐off radius of 8.5Å in Equation (3) from the surface, the highest‐probability actions (HPAs) from all sites are oriented toward the surface. The HPAs from surface adsorption sites point to neighbor surface sites. This policy provides orientation for the hydrogen atoms to migrate across the local energy barriers toward the surface sites. The HPAs from sites close to the surface have larger *Q* ^LSS^ values than that far from the surface, as the discount factor reduces the contribution of long‐term rewards to the *Q* ^LSS^ function compared to short‐term rewards.

We compare the annealing process using RL LSS and the Metropolis–Hastings algorithm,^[^ ^35^ ^]^ as shown in Figure 5b,c. RL LSS leads all hydrogen atoms to surface adsorption sites and converges to the energy ground states in 200 timesteps in all 50 trajectories. From the grey lines, one can observe that the system moves across a large number of low‐energy barriers and approaches the ground state. In comparison, the Metropolis–Hastings algorithm converges slowly. Less than half of the hydrogen migrates to the surface sites in 500 timesteps annealing, leaving ≈4eV energy above the ground state on average. These results demonstrate that the LSS can show advantageous performance in approaching low‐energy configurations compared to straightforward Monte Carlo methods. A long lookahead *t*_horizon_ provides incentive for the hydrogen atoms stuck in the middle to move up, and the transition path networks self‐assembled in Figure 5a look similar to the approach taken in the previous diffusive MD (DMD) algorithm.^[^ ^2^ ^]^ DMD is however a much cruder dynamical simulator, without taking into account the correlations between adjacent atoms, which are now satisfactorily covered by the TEL neural network *Q*_θ_.

## 3 Discussion and Conclusions

A major difference between the training schemes for TKS and LSS is that the TKS only learns the immediate reward: *Q* ^TKS^(*s*_*t*_, *a*_*t*_) → *r*_*t*_, while the LSS learns the cumulative future reward $Q^{\mathrm{LSS}}\left(s_{t},a_{t}\right)\to E\left[\sum_{\tau =t}^{\infty }\gamma^{\tau -t}r_{\tau }\right]$. The reason why we design the learning scheme in this way is that the TKS aims to reproduce the KMC transition probabilities, which depend only on the forward transition rates from the current states. Thus, RL TKS is a contextual bandit problem, and the *Q* function is trained as supervised learning using training dataset iteratively generated by sampling trajectories. In comparison, the LSS aims to solve a global minimization problem to find the low‐energy states. The trajectory toward low‐energy states involves a large number of future transitions (Figure 4), so the selection of action needs to consider which action is more promising in reducing energy in the long term. Optimizing the cumulative future rewards requires training algorithm beyond supervised learning, and the DQN of our choice is one of the well‐developed RL methods to deal with cumulative rewards optimization.^[^ ^33^ ^]^

TKS and LSS can be viewed as two special limits of a unified RL DQN dynamics. The generalized present reward function can be written as a linear combination of the forward barriers and profits:

(13) $$r_{t}=-\alpha \left(\tilde{F}\left(s_{t}^{\text{saddle}}\right)-F\left(s_{t}\right)\right)-\beta \left(F\left(s_{t+1}\right)-F\left(s_{t}\right)\right)$$

where

(14) $$F\left(s\right)\equiv E\left(s\right)+k_{B}T\sum_{i=1}^{3M}\log\nu_{i}\left(s\right)+F_{0}$$

is the vibrational free energy of state *s*, and

(15) $$\tilde{F}\left(s^{\text{saddle}}\right)\equiv E\left(s^{\text{saddle}}\right)+k_{B}T\sum_{j=1}^{3M-1}\log\nu_{j}^{*}\left(s^{\text{saddle}}\right)+F_{0}$$

is the effective free energy of the saddle point *s* ^saddle^ (*F*_0_ is a state‐independent constant). There are three continuously tunable dimensionless parameters (α, β, γ) then in DQN dynamics Equation (1) using the generalized

(16) $$Q\equiv E\left[\sum_{t=0}^{t_{\mathrm{horizon}}-1}\gamma^{t}r_{t}\right]$$

and its learning *Q*_θ_. α, β, γ controls the importance assigned to reproducing the present‐step transition probabilities, present‐step potential energy reductions, and long‐term lookahead of the model, respectively. TKS and LSS correspond to (α = 1, β = 0, γ = 0) and (α = 0, β = 1, γ ≃ 1) corners of the general (α, β, γ) parameter space, respectively. Other parametric settings, for which we are still seeking physical meaning in statistical physics (see Section 4.3), can be used to explore different aspects of PES with certain preferences. A probabilistic interpretation of this general DQN framework equations ((1), (13), (14), (15), (16)) is discussed in Section 4.3, mapping each parameter set to a probability distribution function from which the trajectory is sampled.

Our method provides a general computational framework to simulate the long‐timescale diffusion and annealing process. Although the simulations in this paper focus on hydrogen diffusion in metals, the method is actually applicable to diffusion processes in different materials and microstructures, given a specifically designed action space. This method can also bridge large length scales, by first training a model on varied small structures, then deploying the model to guide the long‐timescale simulation in a large supercell that includes the complexity of all trained structures.

## 4 Method

### 4.1 Action Space Identification Algorithm

A big part of our computational saving comes from the learning of a reduced‐dimension TEL, that is, the energetic forward barriers and profits for a given action. The action space $A\left(s\right)\equiv \{a=\left(i,\vec{v}\right)\}$ is identified based on the atomic configuration *s*. The ground truths for these energetic barriers and profits in the space of actions is computed based on the NEB or other rigorous algorithms navigating the 3*N*‐dimensional potential energy landscape (PES). But once learned, the “transition‐energy landscape” is smaller in dimension (dimA(s)≪dims=3N) and much faster to evaluate than running NEB calculations on the fly. One can also think of A(s) as the equivalent of “attention” mechanism^[^ ^36^ ^]^ in the atomic configuration space, focusing only on the small cluster of atoms that is likely to be altered at present in *s*. The reduced‐dimension TEL is therefore an on‐the‐fly, adaptive data superstructure that are built on top of the well‐known 3*N*‐dimensional PES, represented by our “forward barrier” (Equation (15)) and “profit” (Equation (9)) neural networks for evaluating Equation (13).

The algorithm first identifies all hydrogen atoms with indices *i*_1_, *i*_2_, ⋅⋅⋅. For each hydrogen atom *i*, the distance of all metal atoms *j* within a cut‐off radius *r*_c_ is ranked

(17) $$r_{ij_{1}}\leq r_{ij_{2}}\leq \cdots \leq r_{ij_{M}}$$

where $r_{ij_{k}}$ is the distance between atom *i* and atom *j* (the *k*th nearest neighbor of *i*). Then, we use all metal atoms *j*_*k*_ with a distance $r_{ij_{k}}<1.2r_{ij_{4}}$ (we denote the largest *k* satisfying the condition as *n*) and the hydrogen atom *i* itself to construct a 3D convex hull including these atoms. If the hydrogen atom *i* is a corner of the convex hull, the hydrogen atom is on a surface adsorption site; if the hydrogen atom *i* is inside the convex hull, the hydrogen atom is a bulk interstitial site.

If the hydrogen atom is in a bulk interstitial site, we choose all face centers, $\left({\vec{c}}_{1},{\vec{c}}_{2},\text{…},{\vec{c}}_{m}\right)$, of the convex hull (*j*_1_, ⋅⋅⋅, *j*_*n*_). Then, the actions toward every face center $(i,\max(1.6\left({\vec{c}}_{k}-{\vec{r}}_{i}\right),1.2Å\frac{{\vec{c}}_{k}-{\vec{r}}_{i}}{|{\vec{c}}_{k}-{\vec{r}}_{i}|})),k=1,2,\text{…},m$ are included into the action space, except there are “collisional” events. The “collisional” event is defined as, if the hydrogen atom *i* takes the action, it will have a smaller distance than 0.5 Å with at least one other atom. If the hydrogen atom “collides” with another hydrogen atom, the action is directly discarded. If the hydrogen atom “collides” with a metal atom, the metal atom will be added to reconstruct the convex hull, and actions toward face centers adjacent to the added atom will be included, except if it evokes another “collision.” If that happens, the action will be directly discarded.

If the hydrogen atom is on the surface adsorption site, the convex hull is reconstructed using metal atoms *j*_*k*_ satisfying $r_{ij_{k}}<1.2r_{ij_{3}}$. Atoms directly connected with the hydrogen atom, (*j*_1_, *j*_2_, ⋅⋅⋅, *j*_*n*_), are identified as the adsorption site (we sort (*j*_1_, *j*_2_, ⋅⋅⋅, *j*_*n*_) to form a counter‐clockwise loop). The adsorption site center is obtained as $\vec{c}=\frac{1}{n}\sum_{k}{\vec{r}}_{j_{k}}$. The adsorption site has *n* edges, and the *s*th edge center is ${\vec{e}}_{s}\equiv \left({\vec{r}}_{j_{s}}+{\vec{r}}_{j_{s+1}}\right)/2$. First, the surface diffusion actions $(i,1.6\left({\vec{e}}_{s}-\vec{c}\right)),s=1,2,\text{…},n$ are included. Then, the action toward the bulk $\left(i,3Å\frac{{\vec{c}}_{k}-{\vec{r}}_{i}}{|{\vec{c}}_{k}-{\vec{r}}_{i}|}\right)$ is included. If “collision” happens, the same procedure as the bulk interstitial site case is applied.

### 4.2 Computational Costs Estimation

**Figure 6:**
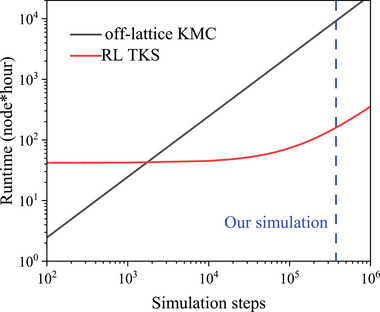
Computation costs estimation of our RL TKS method compared to off‐lattice KMC method without deep learning in the hydrogen diffusion problem in equiatomic CrCoNi medium entropy alloy. RL TKS runtime includes both NN training and simulation running on the PFP online server.

We estimate the computational costs of RL TKS compared to the off‐lattice KMC for hydrogen diffusion in the equiatomic CrCoNi, as shown in **Figure** **6**. The RL training time is 42 node hour, the simulation time per transition step is 1.13 and 88.5 node s for the RL and off‐lattice KMC, respectively. The cross‐point of the two curves is at 1730 simulation steps, and we have 375 000 steps to produce Figure 3. We note that RL TKS is fundamentally equivalent to KMC. The reason for the acceleration in the case of RL TKS is solely because we have used deep learning to construct a reduced‐dimension “transition‐energy landscape” that is super fast to evaluate.

### 4.3 Physical Interpretation of the General DQN Dynamics

The most general form, Equations (1) and (13–(16), in the RL framework, does not produce dynamics identical to that of the physical dynamics of trapped metastable systems, often well approximated by Markovian network^[^ ^37^ ^]^ + HTST in statistical kinetics. However, we feel this general dynamics parameterized by continuous parameters (α, β, γ) should still have certain physical meaning. In this section, we will explore the possible conceptual explanations of RL dynamics, in different regimes of (α, β, γ).

By setting the parameters (α, β, γ), our method samples different time‐dependent probability distributions. In physical reality, the transition rate is approximately determined by the HTST:.

(18) $$\begin{array}{ccc} \Gamma_{s_{t}a_{t}} & = & \nu_{a}e^{-\left(E\left(s_{t}^{\mathrm{saddle}}\right)-E\left(s_{t}\right)\right)/k_{B}T}\\ & = & e^{-\left(\tilde{F}\left(s_{t}^{\mathrm{saddle}}\right)-F\left(s_{t}\right)\right)/k_{B}T} \end{array}$$

If thermal equilibrium is reached (time‐dependent → time‐independent probability distribution), the probability distribution among different states in the state space S is

(19) $$P\left(s\right)=\frac{1}{Z}e^{-F\left(s\right)/k_{B}T},\;\;Z=\sum_{s\in S}e^{-F\left(s\right)/k_{B}T}$$

Below we analyze two different limits of RL discount‐rate γ = 0, where only the forward barrier and profit at present are relevant, and γ ≈ 1, where all future profits within the time horizon *t*_horizon_ are relevant.

#### 4.3.1 γ = 0 and Modified Detailed Balance

If γ = 0, the value function cares only about the present, $Q^{*}\left(s_{t},a_{t}\right)=r_{t}=-\alpha \left(\tilde{F}\left(s_{t}^{\mathrm{saddle}}\right)-F\left(s_{t}\right)\right)-\beta \left(F\left(s_{t+1}\right)-F\left(s_{t}\right)\right)$. The problem simplifies into choosing an action based on the next step reward, namely, a contextual bandit problem. If the parameterized *Q*_θ_(*s*, *a*) properly reproduce the exact value function Q*(*s*, *a*), the policy gives

(20) $$\pi_{\theta }\left(a|s\right)=\frac{{\left(\Gamma_{sa}\right)}^{\alpha }P{\left(s_{sa}^{\prime }\right)}^{\beta }}{\sum_{a^{\prime }\in A_{s}}{\left(\Gamma_{sa^{\prime }}\right)}^{\alpha }P{\left(s_{sa^{\prime }}^{\prime }\right)}^{\beta }}$$

where $s_{sa}^{\prime }$ is the next state after taking action *a*. For RL TKS that reproduces the transition rates of Equation (18), the coefficients are set as α = 1, β = 0. The policy then gives

(21) $$\pi_{\theta }\left(a|s\right)=\frac{\Gamma_{s_{t}a}}{\sum_{a^{\prime }}\Gamma_{s_{t}a^{\prime }}}$$

and the stationary time of the system at state *s*, τ(*s*), is evaluated as

(22) $$\tau \left(s_{t}\right)=\frac{1}{\sum_{a}\Gamma_{sa}}=\frac{1}{\sum_{a}e^{Q^{*}\left(s,a\right)/k_{B}T}}$$

**Figure 7:**
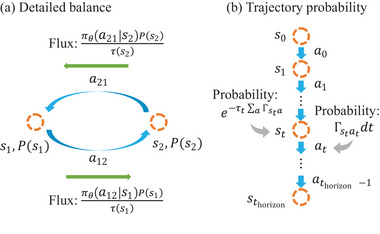
Illustration of the a) detailed balance and b) trajectory probabilities. Orange circles, blue arrows, and green arrows represent states, transitions, and probability flux, respectively.

In certain scenarios, the goal is to sample thermal equilibrium distribution. The detailed balance principle (**Figure** **7**a) states that if the following kinetic laws holds for arbitrary states *s*_1_, *s*_2_

(23) $$\frac{1}{\tau (s_{1})}\pi_{\theta }\left(a_{12}|s_{1}\right)P\left(s_{1}\right)=\frac{1}{\tau (s_{1})}\pi_{\theta }\left(a_{21}|s_{2}\right)P\left(s_{2}\right)$$

then the sampled states will follow the thermal equilibrium distribution *P*(*s*), as given in Equation (19). Here, *a*_*ij*_ means the action of transition from state *s*_*i*_ to state *s*_*j*_. Equation (23) is equivalent to:

(24) $$\exp\left\{\frac{Q^{*}\left(s_{1},a_{12}\right)-Q^{*}\left(s_{2},a_{21}\right)}{k_{B}T}\right\}=\exp\left\{-\frac{F\left(s_{2}\right)-F\left(s_{1}\right)}{k_{B}T}\right\}$$

the well‐known forward barrier–backward barrier–thermodynamics connection. As $Q^{*}\left(s_{i},a_{ij}\right)=-\alpha \left(\tilde{F}\left(s^{\mathrm{saddle}}\right)-F\left(s_{i}\right)\right)-\beta \left(F\left(s_{j}\right)-F\left(s_{i}\right)\right)$, we have

(25) $$Q^{*}\left(s_{1},a_{12}\right)-Q^{*}\left(s_{2},a_{21}\right)=\left(\alpha +2\beta \right)\left(F\left(s_{1}\right)-F\left(s_{2}\right)\right)$$

Then standard detailed balance Equation (24) would demand

(26) α+2β=1

So we proved that the steady‐state probability distribution can approach Equation (19) as long as α + 2β = 1.

But interestingly, if α + 2β ≠ 1, an altered form of detailed balance still holds.

(27) $$\exp\left\{\frac{Q^{*}\left(s_{1},a_{12}\right)-Q^{*}\left(s_{2},a_{21}\right)}{k_{B}T}\right\}=\exp\left\{-\frac{F\left(s_{2}\right)-F\left(s_{1}\right)}{k_{B}T_{\mathrm{eq}}}\right\}$$

just with

(28) $$T_{\mathrm{eq}}\;\equiv \frac{T}{\alpha +2\beta }$$

In other words, γ = 0 DQN can have two temperatures, a kinetic temperature *T* using which we run Equation (1), and a thermodynamic temperature *T*_eq_ where, when steady state is approached, the probability distribution still has a canonical form (Equation (19)) but with a rescaled temperature $T_{\mathrm{eq}}=\frac{T}{\alpha +2\beta }$. So if α + 2β > 1, the thermodynamic temperature is lower than the kinetic temperature, and vice versa.

Algorithmically we do not need to always keep a constant α, β. For example, one strategy could be while keeping *T*_eq_ constant, one varies β/α, and a larger β/α ratio could promote sampling more rare transition events while keeping the eventual thermodynamic properties correct. Indeed, if α → 0, we would be doing classical Metropolis Monte Carlo^[^ ^38^ ^]^ (MC, not KMC) for sampling the equilibrium thermodynamic distribution. Thus, β/α is a knob to smoothly tune from KMC to MC.

Because detailed balance is such an important concept in statistical kinetics (to prevent, e.g., infinite looping between a ring of states at thermodynamic equilibrium), it is important to discuss about the type of numerical errors that could break detailed balance. Due to the neural network prediction error, *Q*_θ_ and Q* are not *exactly* the same, and may contain numerical noise. If we approximate Q* by *Q*_θ_ in Equation (25), the detailed balance Equation (27) may not hold exactly. Therefore, the neural network prediction error will influence reaching thermodynamic equilibrium *T*_eq_.

If exact detailed balance is desirable, one can do the following “symmetrization procedure” with a higher computational cost: for each *Q*_θ_(*s*_*i*_, *a*_*ij*_), we apply the action and get the next state *s*_*j*_, and calculate *F*(*s*_*j*_) and the backward *Q*_θ_(*s*_*j*_, *a*_*ji*_), returning to *i*. We then use the symmetrized value function

(29) $$\begin{array}{ccc} & & Q_{\theta }^{\mathrm{corrected}}\left(s_{i},a_{ij}\right)\;\equiv \;Q_{\theta }\left(s_{i},a_{ij}\right)+\\ & & \frac{Q_{\theta }\left(s_{j},a_{ji}\right)-Q_{\theta }\left(s_{i},a_{ij}\right)-\left(\alpha +2\beta \right)\left(F\left(s_{j}\right)-F\left(s_{i}\right)\right)}{2} \end{array}$$

always to sample the action. This ensures detailed balance and reaching thermodynamic equilibrium *T*_eq_ despite of neural network error.

#### 4.3.2 γ ∼ 1: Maximizing the Path Probability of a Trajectory

When we set γ ∼ 1, the algorithm maximizes the total reward of the trajectory $J\equiv (s_{0},a_{0},\tau_{0},s_{1},a_{1},\tau_{1},\text{…}s_{t_{\mathrm{horizon}}})$ (Figure 7b), $R\left(J\right)≃\sum_{t=0}^{t_{\mathrm{horizon}}}r_{t}$ (*t*_horizon_ is the time horizon of the trajectory. We consider setting γ slightly smaller than 1 as a convergence technique that leads to a small bias).

The physical probability of a trajectory J according to the conventional Markovian network^[^ ^37^ ^]^ + HTST in standard statistical kinetics, given an initial state *s*_0_, is a product of two factors: 1) the probability of staying in state *s*_*t*_ for physical time τ_*t*_ equals $e^{-\tau_{t}\sum_{a}\Gamma_{s_{t}a}}$, and 2) the probability of the transition from *s*_*t*_ to *s*_*t* + 1_ in a small time interval $\left[\sum_{i=0}^{t}\tau_{i},\;d\tau +\sum_{i=0}^{t}\tau_{i}\right]$ equals $\Gamma_{s_{t}a_{t}}d\tau$. We multiply all factors together to get the total probability of getting the trajectory J as

(30) $$P\left(J|s_{0}\right)=\prod_{t=0}^{t_{\mathrm{horizon}}-1}e^{-\tau_{t}\sum_{a}\Gamma_{s_{t}a}}\Gamma_{s_{t}a_{t}}d\tau$$

In any trajectory that our DQN method outputs, the expected stationary time $\tau_{t}=1/\sum_{a}\Gamma_{s_{t}a}$, so $e^{-\tau_{t}\sum_{a}\Gamma_{s_{t}a}}=e^{-1}$ and the probability product becomes $P\left(J|s_{0}\right)=\prod_{t=0}^{t_{\mathrm{horizon}}-1}\Gamma_{s_{t}a_{t}}\left(e^{-t_{\mathrm{horizon}}}d\tau^{t_{\mathrm{horizon}}}\right)$. As $e^{-t_{\mathrm{horizon}}}d\tau^{t_{\mathrm{horizon}}}$ is a constant independent from the policy, we can write the path probability as

(31) $$P\left(J|s_{0}\right)\propto \exp\left\{-\sum_{t=0}^{t_{\mathrm{horizon}}-1}\frac{\tilde{F}\left(s_{t}^{\mathrm{saddle}}\right)-F\left(s_{t}\right)}{k_{B}T}\right\}$$

according to the conventional Markovian chain + HTST. Equation (31) is in fact a path integral akin to the action integral of a trajectory in classical mechanics, and the “principle of least action” applies when we think about the most likely physical trajectory of a metastable system on a Markovian network.^[^ ^37^ ^]^

In contrast, in our general DQN trajectory, the total reward can be rewritten as:

(32) $$\begin{array}{cc} R(J) & =-\alpha \sum_{t=0}^{t_{\mathrm{horizon}}-1}\left(\tilde{F}\left(s_{t}^{\mathrm{saddle}}\right)-F\left(s_{t}\right)\right)\\ & \;\;-\beta \sum_{t=0}^{t_{\mathrm{horizon}}-1}\left(F\left(s_{t+1}\right)-F\left(s_{t}\right)\right)\\ & =k_{B}T\left[\alpha \log P(J|s_{0})+\beta \log P(s_{t_{\mathrm{horizon}}})\right]+C_{0} \end{array}$$

where *C*_0_ is a constant independent from the policy. So we can see that maximizing the total rewards in DQN with γ ∼ 1 is equivalent to maximizing

(33) $$A\equiv \alpha \log P(J|s_{0})+\beta \log P(s_{t_{\mathrm{horizon}}}).$$

We can see right away that while this is different from the physical action integral (31), it does contain the path‐integral contribution, while also mixing with the final energy drop $F\left(s_{t_{\mathrm{horizon}}}\right)-F\left(s_{0}\right)$. So the physical interpretation of α is an emphasis on “procedural justice,” while β emphasizes “the end justifies the means” (consider that Metropolis MC^[^ ^38^ ^]^ has only β and not α, while KMC requires only α and not β).

If α = 0, β = 1, the method aims to sample the most probable final state $s_{t_{\mathrm{horizon}}}$ were the system at thermal equilibrium, corresponding to an annealing process that targets the ground state. If on the one hand α = 1, β = 0, the method aims to sample the most probable trajectory based on transition kinetics. In the most general case, α and β can be tuned to balance “procedural justice” with “the end justifies the means.”

Again, α, β and even γ only need to be piece‐wise constant, and the relative emphasis on “procedural justice” versus “end justifies the means” may be tuned on the fly. For example, one could first use large β/α and γ to scope out the possible global direction of free‐energy reduction (see Figure 5), perform on‐the‐fly training of the relevant forward barriers and profits, and then based on this experience, downtune the β/α as well as γ to get more and more realistic physical time estimation of the paths in this general direction. In other words, one may engineer “morphing” of the RL dynamics in (α, β, γ) parameter space, from the (0, 1, 1^−^) corner running long time horizon annealing, to the (1^−^, 0^+^, 0^+^) corner (KMC) running physical timescale kinetics.

## Conflict of Interest

The authors declare no conflict of interest.

## Supporting information

Supporting Information

## Data Availability

The data that support the findings of this study are available from the corresponding author upon reasonable request.
