# Supplementary material for: Reinforcement Learning‐Guided Long‐Timescale Simulation of Hydrogen Transport in Metals
Source: Adv Sci (Weinh). 2023 Dec 7;11(5):2304122. doi: 10.1002/advs.202304122 (PMC10837361; doi:10.1002/advs.202304122)
Supplement: Supplementary file 1 — Supporting Information [file ADVS-11-2304122-s001.pdf]

ADVANCED SCIENCE
Open Access

Supporting Information

for *Adv. Sci.*, DOI 10.1002/advs.202304122

Reinforcement Learning-Guided Long-Timescale Simulation of Hydrogen Transport in Metals

*Hao Tang, Boning Li, Yixuan Song, Mengren Liu, Haowei Xu, Guoqing Wang, Heejung Chung and Ju Li**

Suporting Information: reinforcement learning-guided long-timescale simulation of hydrogen transport in metals

Hao Tang,[1] Boning Li,[2,3] Yixuan Song,[1] Mengren Liu,[1] Haowei Xu,[4] Guoqing Wang,[2,4] Heejung Chung,[1] and Ju Li[1,4,*]
[1]*Department of Materials Science and Engineering, Massachusetts Institute of Technology, MA 02139, USA*
[2]*Research Laboratory of Electronics, Massachusetts Institute of Technology, Cambridge, MA 02139, USA*
[3]*Department of Physics, Massachusetts Institute of Technology, MA 02139, USA*
[4]*Department of Nuclear Science and Engineering, Massachusetts Institute of Technology, Cambridge, MA 02139, USA*
(Dated: November 22, 2023)

S1. NUMERICAL DETAILS OF ATOMISTIC SIMULATION AND RL TRAINING

The model training on pure copper and nickel is conducted on $4 \times 4 \times 4$ cubic supercell of the FCC metals. 3 atomic configurations are generated for each metal, where 4 hydrogen atoms are randomly sampled in all octahedral and tetrahedral sites in each configuration. 20 and 40 trajectories are sampled for copper and nickel, respectively, with 30 timesteps in each. In the atomic relaxation and NEB calculations, all forces converge to 0.05 eV/Å under the PreFerred Potential (PFP) v4.0.0, which is used throughout this paper. The cut-off radius of the neural network model is 4 Å. The embedding network G^1_k has one hidden layer and an output layer both with a size of 12. Throughout the paper, we take the first 1/4 columns of G^1_k to form G^2_k, and the input layers of $G^{1,2}_k$ have a size of $N_c + 1$, where N_c is the number of chemical species. We define an element species list: $C = (C_1, C_2, \cdots, C_{N_c}, C_{N_c+1} = \text{action})$, where C_l is the lth element. For $G^{1,2}_k(f_c(r_{im}), c_m = C_l)$, the input layer takes the N_c+1 dimensional input vector whose lth component is $f_c(r_{im})$ and other components are zeros. The fitting network has two hidden layers with a size of 32. The maximum atom number (within the cut-off radius of each atom) of the "transition energy landscape" is set as 40, which has not been exceeded during the training. The training temperature is set as 1000 K throughout this paper. After including the nth trajectory, one randomly samples a trajectory from probability distribution $P_i = \frac{1-0.99}{1-0.99^n} 0.99^{n-i}$ (recent trajectory has larger probability) and train 20 gradient descend steps from the sampled trajectory, and repeat this for n times. The training algorithm is Adam throughout this paper, and the learning rate here is set as 10^{-3} in all online training. Offline training is conducted to further improve the model's accuracy. We separate the training data into the training dataset (2/3 of the data) and the testing dataset (1/3 of the data). 10000 full gradient descent is implemented on the training dataset. The learning rate changes from 10^{-3} to 10^{-5} that exponentially decays with timesteps in all offline training in this paper.

The model training on NiCrCo medium entropy alloy is conducted on $4 \times 4 \times 4$ cubic supercell of the FCC fully random solid solution. 9 atomic configurations are generated for each metal, where 4 hydrogen atoms are randomly sampled in all octahedral and tetrahedral sites in each configuration. 3 independent processes of training are conducted with 101 trajectories in each, and each trajectory contains 30 timesteps. In the atomic relaxation and NEB calculations, all forces converge to 0.05 and 0.07 eV/Å, respectively. The cut-off radius of the neural network model is 5 Å. The embedding network G^1_k has one hidden layer and an output layer both with a size of 24. The fitting network has two hidden layers with a size of 128. The maximum atom number is set as 50, which was not exceeded during the training. The online training parameters are the same as pure metals. As to offline training, we separate the training data the same way as pure metals. Stochastic gradient descent is implemented with a minibatch size of 500 data points (one timestep is a data point). The minibatch is randomly sampled from all data points, and 10 gradient descent steps are applied to each minibatch. That is repeated for 20000 iterations. In order to avoid overfitting, a normalization term of $5 \times 10^{-6} \parallel \theta \parallel^2$ is added to the loss function.

The deep Q-network learning for copper (111) surface is conducted on $4 \times 4 \times 3$ hexagonal lattice of FCC copper (4 replications on a and b directions and 3 replications on c direction. c direction is along the 3-fold axis). A vacuum layer of 15 Å is included in the c direction. We implemented 7 independent training processes, 4 of them have only one randomly sampled hydrogen atom in the copper slab (12 configurations are sampled as starting points, and initial configurations are randomly selected from them), and the other 3 have 10 randomly sampled hydrogen atoms (10 configurations are sampled as starting points). 300 trajectories are sampled with 30 timesteps in each. In the atomic relaxation, all forces converge to 0.05 eV/Å. The cut-off radius of the neural network model is 8.5 Å, as the model needs more distant atomic information to foresee the long-term rewards. The embedding network G^1_k has one hidden layer and an out-

* liju@mit.edu

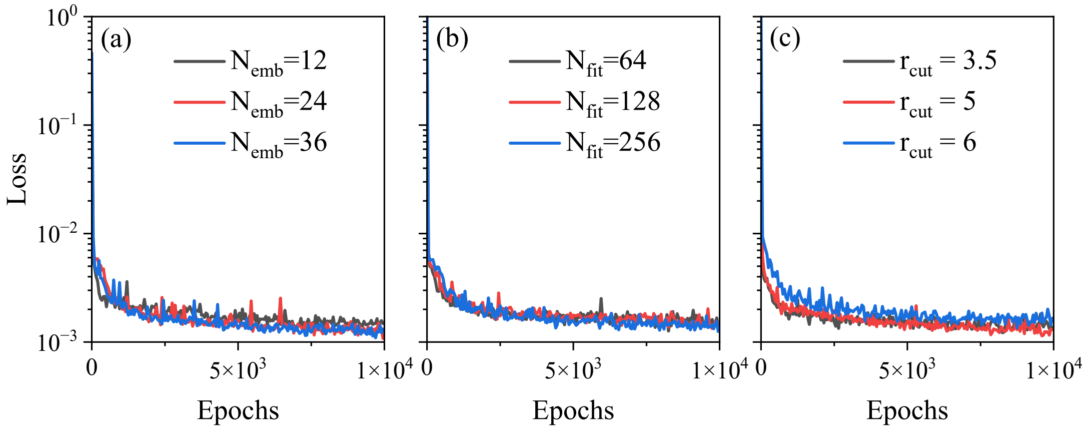

FIG. S1. Training curve (loss *vs* training epochs) of the TKS NN model for the hydrogen diffusion in equiatomic CrCoNi using different model hyperparameters. The N_{emb}, N_{fit}, and r_{cut} are the width of embedding network, fitting network, and the cut-off radius, respectively. We use $N_{\mathrm{emb}} = 24, N_{\mathrm{fit}} = 128, r_{\mathrm{cut}} = 5$ Å for unlabeled parameters in each panel.

put layer both with a size of 24. The fitting network has two hidden layers with a size of 128. The maximum atom number is set as 260, which has not been exceeded during the training. After including the nth trajectory, one randomly samples a trajectory and trains 5 gradient descent steps from the sampled trajectory, and repeats this for $\lceil n^{2/3} \rceil$ times. The offline training randomly samples a mini-batch with 10 trajectories and applies 10 steps of gradient descent at each iteration. There are 1010 iterations in the training process.

S2. NEURAL NETWORK TRAINING PARAMETERS

In this section, we compare the training curves using different neural network hyperparameters to validate our choice of the NN hyperparameter settings. We use hydrogen diffusion in equiatomic CrCiNi as an example, as shown in Fig. S1. For the embedding network width N_{emb}, our choice $N_{\mathrm{emb}} = 24$ gives similar training loss with $N_{\mathrm{emb}} = 36$, slightly better than $N_{\mathrm{emb}} = 12$ (Fig. S1a). The fitting network width $N_{\mathrm{fit}} = 64, 128, 256$ gives similar training loss (Fig. S1b). For the cut-off radius r_{cut}, our choice $r_{\mathrm{cut}} = 5$ gives the lowest training lost within $r_{\mathrm{cut}} = 3.5, 5, 6$ (Fig. S1c). In all plots, the loss function converges with respect to epochs. The tests validate that our NN settings give close-to-convergent model performance.

If one wants to further improve the model performance, a more sophisticated NN architecture design will be necessary. A promising choice is to use equivariant graph neural networks [1–3] to represent the $Q_\theta(s, a)$ function, where the state s is represented by a graph and the action a is represented by a vector input on nodes.

[1] S. Takamoto, S. Izumi, and J. Li, Comput. Mater. Sci. **207**, 111280 (2022).
[2] S. Takamoto, D. Okanohara, Q. Li, and J. Li, J. Materiomics **9**, 447 (2023).
[3] S. Batzner, A. Musaelian, L. Sun, M. Geiger, J. P. Mailoa, M. Kornbluth, N. Molinari, T. E. Smidt, and B. Kozinsky, Nature communications **13**, 1 (2022).
